# Signor Della Fanteria on the Reunion of Two Fingers, &c.

**Published:** 1842-10-01

**Authors:** 


*Reunion of Two Fingers completely separated from the Hand, and each divided into two portions.* By SIGNOR DELLA FANTERIA.—A girl, 14 years old, was engaged with another person in some domestic occupation, when the latter accidentally let fall a knife, which cut off two of her fingers below the first phalanx. The author being soon after summoned, found the two pieces in some meal on which the patient's hand was resting at the time of the accident; but he discovered, to his great surprise, that each of them was divided into two portions. However, he determined to try to unite them; and having put the bits together, he kept them all in their places with sutures and strips of plaster. In a few days the adhesion was completed, and the patient ultimately recovered the entire use of her fingers.

It is necessary to mention, that the authenticity of this strange case was confirmed by Professors Centofanti and Vacca.—*London Med. Gaz.* July 22, 1842. *From Ann. Univ. di Med.*

